# Crystal structure of (eth­oxy­ethyl­idene)di­methyl­aza­nium ethyl sulfate

**DOI:** 10.1107/S2056989015020678

**Published:** 2015-11-07

**Authors:** Ioannis Tiritiris, Stefan Saur, Willi Kantlehner

**Affiliations:** a Fakultät Chemie/Organische Chemie, Hochschule Aalen, Beethovenstrasse 1, D-73430 Aalen, Germany

**Keywords:** crystal structure, (eth­oxy­ethyl­idene)di­methyl­aza­nium, ethyl sulfate, salt, hydrogen bonding

## Abstract

In the title salt, C_6_H_14_NO^+^·C_2_H_5_SO_4_^−^, the C—N bond lengths in the cation are 1.2981 (14), 1.4658 (14) and 1.4707 (15) Å, indicating double- and single-bond character, respectively. The C—O bond length of 1.3157 (13) Å shows double-bond character, indicating charge delocalization within the NCO plane of the iminium ion. In the crystal, C—H⋯O hydrogen bonds between H atoms of the cations and O atoms of neighbouring ethyl sulfate anions are present, generating a three-dimensional network.

## Related literature   

For the crystal structure of l-argininium ethyl sulfate, see: Karapetyan (2008 ▸). For the crystal structure of (meth­oxy­methyl­idene)di­methyl­aza­nium tetra­phenyl­borate aceto­nitrile monosolvate, see: Tiritiris *et al.* (2014*a* ▸). For the crystal structure of (but­oxy­methyl­idene)di­methyl­aza­nium tetra­phenyl­borate aceto­nitrile monosolvate, see: Tiritiris *et al.* (2014*b* ▸).
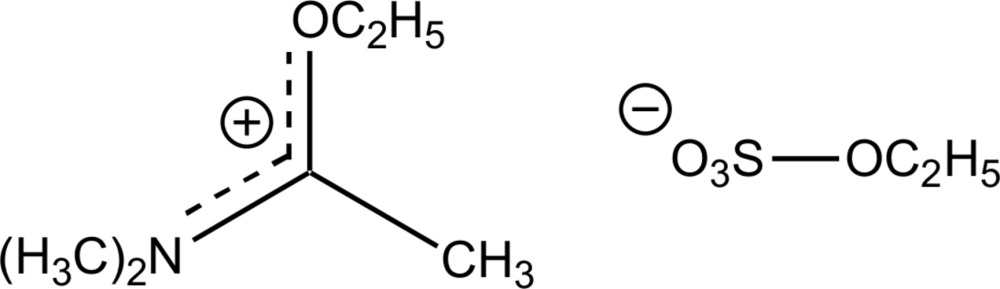


## Experimental   

### Crystal data  

C_6_H_14_NO^+^·C_2_H_5_O_4_S^−^
*M* *_r_* = 241.30
Monoclinic, 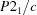

*a* = 13.3979 (8) Å
*b* = 7.2860 (4) Å
*c* = 12.5284 (8) Å
β = 97.712 (3)°
*V* = 1211.92 (13) Å^3^
*Z* = 4
Mo *K*α radiation
μ = 0.27 mm^−1^
*T* = 100 K
0.28 × 0.22 × 0.05 mm

### Data collection  

Bruker Kappa APEXII DUO diffractometer
Absorption correction: multi-scan (Blessing, 1995 ▸) *T*_min_ = 0.704, *T*_max_ = 0.746
25216 measured reflections
3734 independent reflections
3142 reflections with *I* > 2σ(*I*)
*R*_int_ = 0.028

### Refinement  

*R*[*F* ^2^ > 2σ(*F* ^2^)] = 0.032
*wR*(*F* ^2^) = 0.091
*S* = 1.05
3737 reflections
142 parameters
H-atom parameters constrained
Δρ_max_ = 0.37 e Å^−3^
Δρ_min_ = −0.42 e Å^−3^

### 

Data collection: *APEX2* (Bruker, 2008 ▸); cell refinement: *SAINT* (Bruker, 2008 ▸); data reduction: *SAINT*; program(s) used to solve structure: *SHELXS97* (Sheldrick, 2008 ▸); program(s) used to refine structure: *SHELXL97* (Sheldrick, 2008 ▸); molecular graphics: *DIAMOND* (Brandenburg & Putz, 2005 ▸); software used to prepare material for publication: *SHELXL97*.

## Supplementary Material

Crystal structure: contains datablock(s) I, global. DOI: 10.1107/S2056989015020678/hb7521sup1.cif

Structure factors: contains datablock(s) I. DOI: 10.1107/S2056989015020678/hb7521Isup2.hkl

Supporting information file. DOI: 10.1107/S2056989015020678/hb7521Isup3.cml

**Fig. 1.**
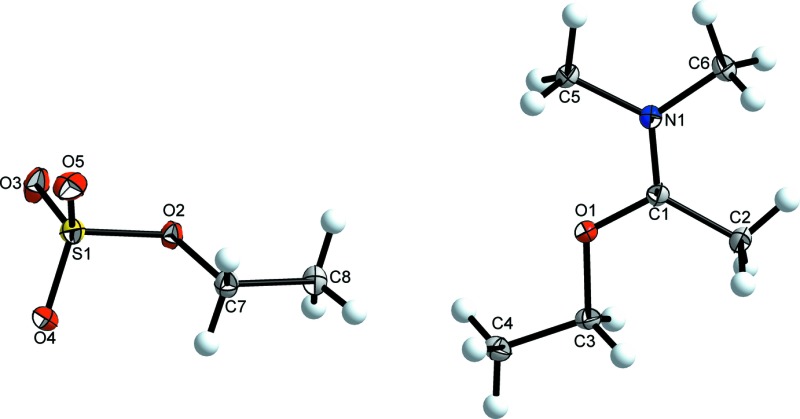
The structure of the title compound with displacement ellipsoids at the 50% probability level.

**Fig. 2.**
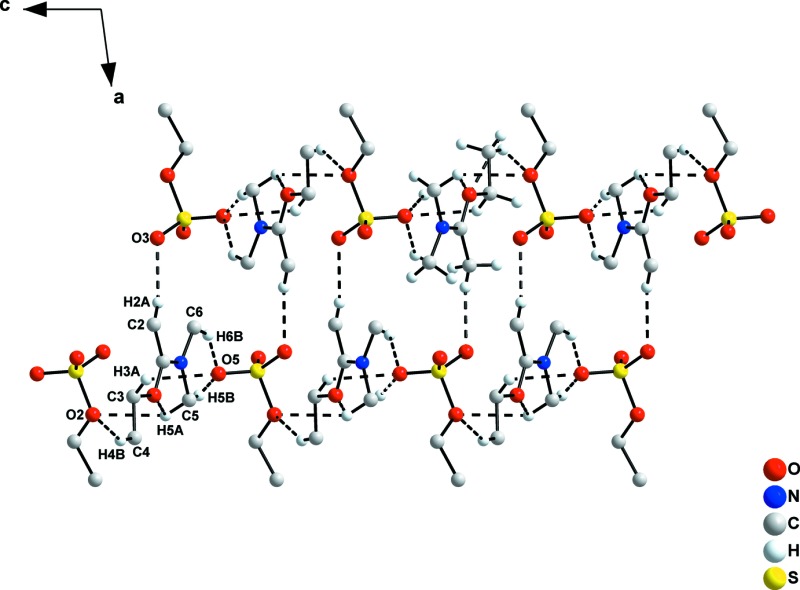
C—H···O hydrogen bonds (black dashed lines) between H atoms of the cations and oxygen atoms of the ethyl sulfate ions (ac view).

. DOI: 10.1107/S2056989015020678/hb7521fig1.tif
The structure of the title compound with displacement ellipsoids at the 50% probability level.

ac . DOI: 10.1107/S2056989015020678/hb7521fig2.tif
C—H⋯O hydrogen bonds (black dashed lines) between H atoms of the cations and oxygen atoms of the ethyl sulfate ions (*ac* view).

CCDC reference: 1434493

Additional supporting information:  crystallographic information; 3D view; checkCIF report

## Figures and Tables

**Table 1:** Hydrogen-bond geometry (Å, °)

*D*—H⋯*A*	*D*—H	H⋯*A*	*D*⋯*A*	*D*—H⋯*A*
C5—H5*B*⋯O5^i^	0.98	2.32	3.288 (2)	170
C2—H2*A*⋯O3^ii^	0.98	2.40	3.341 (2)	161
C3—H3*A*⋯O5	0.99	2.50	3.121 (2)	120
C5—H5*A*⋯O2^iii^	0.98	2.51	3.372 (2)	147
C6—H6*B*⋯O5^i^	0.98	2.54	3.453 (2)	154
C4—H4*B*⋯O2^iv^	0.98	2.55	3.452 (2)	154

Symmetry codes: (i) 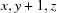; (ii) 
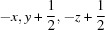
; (iii) 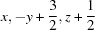; (iv) 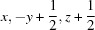.

## supplementary crystallographic information

### S1. Comment

The cation in the title compound is similar to the cations in the structurally known compounds (methoxymethylidene)dimethylazanium tetraphenylborate acetonitrile monosolvate (Tiritiris *et al.*, 2014*a*) and (butoxymethylidene)dimethylazanium tetraphenylborate acetonitrile monosolvate (Tiritiris *et al.*, 2014*b*). According to the structure analysis, the C5–N1 bond length is 1.4658 (14) Å, C6–N1 = 1.4707 (15) Å and C1–N1 = 1.2981 (14) Å, showing single and double bond character, respectively. The C–N1–C angles are: 116.88 (9)° (C5–N1–C6), 120.66 (10)° (C1–N1–C5) and 122.44 (10)° (C1–N1–C6), which indicates a nearly trigonal-planar surrounding of the nitrogen centre by the carbon atoms (Fig. 1). The C–O bond length shows with 1.3157 (13) Å double bond character. The positive charge is completely delocalized on the plane formed by the atoms N1, C1 and O1 (Fig. 1). The bond lengths and angles in the ethyl sulfate ion are in good agreement with the data from the crystal structure analysis of *L*-argininium ethyl sulfate (Karapetyan, 2008). In the crystal structure, C—H···O hydrogen bonds between H atoms of cations and oxygen atoms of neighboring ethyl sulfate ions are present [*d*(H···O) = 2.32–2.55 Å] (Tab. 1), generating a three-dimensional network (Fig. 2).

### S2. Experimental

The title compound was obtained by reacting equimolar amounts of *N*,*N*-dimethylacetamide with diethyl sulfate at room temperature forming (ethoxyethylidene)dimethylazanium ethyl sulfate in nearly quantitative yield. The title compound crystallized after prolonged stay for several years at 273 K, forming colorless single crystals suitable for X-ray analysis.

Diethyl sulfate is carcinogenic, mutagenic and highly poisonous. During the use appropriate precautions must be taken.

### S3. Refinement

The hydrogen atoms of the methyl groups were allowed to rotate with a fixed angle around the C–N and C–C bonds to best fit the experimental electron density, with *U*_iso_(H) set to 1.5*U*_eq_(C) and d(C—H) = 0.98 Å. The H atoms in CH_2_ groups were placed in calculated positions with d(C—H) = 0.99 Å and refined using riding model, with *U*(H) set to 1.2 *U*_eq_(C).

### Crystal data

**Table:**

C_6_H_14_NO^+^·C_2_H_5_O_4_S^−^	*F*(000) = 520
*M**_r_* = 241.30	*D*_x_ = 1.322 Mg m^−^^3^
Monoclinic, *P*2_1_/*c*	Mo *K*α radiation, λ = 0.71073 Å
Hall symbol: -P 2ybc	Cell parameters from 3142 reflections
*a* = 13.3979 (8) Å	θ = 1.5–30.7°
*b* = 7.2860 (4) Å	µ = 0.27 mm^−^^1^
*c* = 12.5284 (8) Å	*T* = 100 K
β = 97.712 (3)°	Plate, colorless
*V* = 1211.92 (13) Å^3^	0.28 × 0.22 × 0.05 mm
*Z* = 4	

### Data collection

**Table:**

Bruker Kappa APEXII DUO diffractometer	3734 independent reflections
Radiation source: fine-focus sealed tube	3142 reflections with *I* > 2σ(*I*)
Triumph monochromator	*R*_int_ = 0.028
φ scans and ω scans	θ_max_ = 30.7°, θ_min_ = 1.5°
Absorption correction: multi-scan (Blessing, 1995)	*h* = −18→19
*T*_min_ = 0.704, *T*_max_ = 0.746	*k* = −10→10
25216 measured reflections	*l* = −17→17

### Refinement

**Table:**

Refinement on *F*^2^	Secondary atom site location: difference Fourier map
Least-squares matrix: full	Hydrogen site location: inferred from neighbouring sites
*R*[*F*^2^ > 2σ(*F*^2^)] = 0.032	H-atom parameters constrained
*wR*(*F*^2^) = 0.091	*w* = 1/[σ^2^(*F*_o_^2^) + (0.0453*P*)^2^ + 0.4489*P*] where *P* = (*F*_o_^2^ + 2*F*_c_^2^)/3
*S* = 1.05	(Δ/σ)_max_ < 0.001
3737 reflections	Δρ_max_ = 0.37 e Å^−^^3^
142 parameters	Δρ_min_ = −0.42 e Å^−^^3^
0 restraints	Extinction correction: *SHELXL97* (Sheldrick, 2008), Fc^*^=kFc[1+0.001xFc^2^λ^3^/sin(2θ)]^-1/4^
Primary atom site location: structure-invariant direct methods	Extinction coefficient: 0.0079 (12)

### Special details

**Table:**

Geometry. All e.s.d.'s (except the e.s.d. in the dihedral angle between two l.s. planes) are estimated using the full covariance matrix. The cell e.s.d.'s are taken into account individually in the estimation of e.s.d.'s in distances, angles and torsion angles; correlations between e.s.d.'s in cell parameters are only used when they are defined by crystal symmetry. An approximate (isotropic) treatment of cell e.s.d.'s is used for estimating e.s.d.'s involving l.s. planes.
Refinement. Refinement of *F*^2^ against ALL reflections. The weighted *R*-factor *wR* and goodness of fit *S* are based on *F*^2^, conventional *R*-factors *R* are based on *F*, with *F* set to zero for negative *F*^2^. The threshold expression of *F*^2^ > σ(*F*^2^) is used only for calculating *R*-factors(gt) *etc*. and is not relevant to the choice of reflections for refinement. *R*-factors based on *F*^2^ are statistically about twice as large as those based on *F*, and *R*- factors based on ALL data will be even larger.

### Fractional atomic coordinates and isotropic or equivalent isotropic displacement parameters (Å^2^)

**Table:**

	*x*	*y*	*z*	*U*_iso_*/*U*_eq_	
O1	0.26034 (6)	0.67627 (11)	0.47160 (7)	0.01926 (17)	
N1	0.17582 (7)	0.90509 (13)	0.38585 (8)	0.01829 (19)	
C1	0.17248 (8)	0.74789 (15)	0.43400 (9)	0.0176 (2)	
C2	0.07696 (9)	0.65143 (17)	0.44583 (10)	0.0231 (2)	
H2A	0.0200	0.7346	0.4257	0.035*	
H2B	0.0778	0.6126	0.5208	0.035*	
H2C	0.0700	0.5435	0.3987	0.035*	
C3	0.26376 (9)	0.50086 (15)	0.52879 (10)	0.0216 (2)	
H3A	0.2195	0.4099	0.4871	0.026*	
H3B	0.2411	0.5165	0.6003	0.026*	
C4	0.37134 (10)	0.43818 (17)	0.54103 (11)	0.0272 (3)	
H4A	0.3920	0.4200	0.4697	0.041*	
H4B	0.3779	0.3222	0.5810	0.041*	
H4C	0.4144	0.5313	0.5804	0.041*	
C5	0.27260 (9)	0.99326 (15)	0.37648 (10)	0.0208 (2)	
H5A	0.3126	1.0010	0.4478	0.031*	
H5B	0.2607	1.1170	0.3468	0.031*	
H5C	0.3092	0.9207	0.3285	0.031*	
C6	0.08461 (9)	1.00066 (18)	0.33605 (11)	0.0276 (3)	
H6A	0.0463	0.9198	0.2830	0.041*	
H6B	0.1038	1.1122	0.3001	0.041*	
H6C	0.0430	1.0336	0.3918	0.041*	
S1	0.19729 (2)	0.47672 (4)	0.17775 (2)	0.01768 (8)	
O2	0.31092 (6)	0.47791 (12)	0.14749 (7)	0.02197 (18)	
O3	0.14783 (7)	0.35675 (14)	0.09598 (8)	0.0313 (2)	
O4	0.16386 (7)	0.66526 (12)	0.17017 (8)	0.02607 (19)	
O5	0.20491 (8)	0.40445 (13)	0.28581 (8)	0.0308 (2)	
C7	0.38088 (9)	0.59962 (17)	0.20875 (10)	0.0228 (2)	
H7A	0.3890	0.5656	0.2859	0.027*	
H7B	0.3561	0.7276	0.2014	0.027*	
C8	0.48008 (9)	0.58239 (19)	0.16523 (12)	0.0288 (3)	
H8A	0.5030	0.4547	0.1714	0.043*	
H8B	0.5302	0.6618	0.2067	0.043*	
H8C	0.4715	0.6196	0.0894	0.043*	

### Atomic displacement parameters (Å^2^)

**Table:**

	*U*^11^	*U*^22^	*U*^33^	*U*^12^	*U*^13^	*U*^23^
O1	0.0167 (4)	0.0153 (3)	0.0259 (4)	−0.0009 (3)	0.0038 (3)	0.0019 (3)
N1	0.0155 (4)	0.0179 (4)	0.0215 (4)	−0.0004 (3)	0.0028 (3)	−0.0003 (3)
C1	0.0167 (5)	0.0174 (5)	0.0191 (5)	−0.0014 (4)	0.0040 (4)	−0.0035 (4)
C2	0.0169 (5)	0.0228 (5)	0.0302 (6)	−0.0047 (4)	0.0050 (4)	−0.0015 (4)
C3	0.0244 (5)	0.0165 (5)	0.0241 (5)	−0.0020 (4)	0.0037 (4)	0.0034 (4)
C4	0.0267 (6)	0.0207 (5)	0.0330 (6)	0.0025 (5)	0.0001 (5)	0.0041 (5)
C5	0.0179 (5)	0.0196 (5)	0.0253 (5)	−0.0024 (4)	0.0047 (4)	0.0029 (4)
C6	0.0188 (5)	0.0264 (6)	0.0365 (7)	0.0031 (4)	0.0000 (5)	0.0062 (5)
S1	0.01773 (13)	0.01682 (13)	0.01937 (13)	0.00004 (9)	0.00569 (9)	−0.00045 (9)
O2	0.0169 (4)	0.0233 (4)	0.0267 (4)	−0.0015 (3)	0.0065 (3)	−0.0075 (3)
O3	0.0238 (4)	0.0363 (5)	0.0346 (5)	−0.0081 (4)	0.0076 (4)	−0.0156 (4)
O4	0.0233 (4)	0.0194 (4)	0.0363 (5)	0.0051 (3)	0.0069 (4)	0.0039 (3)
O5	0.0379 (5)	0.0309 (5)	0.0250 (4)	−0.0011 (4)	0.0090 (4)	0.0098 (4)
C7	0.0195 (5)	0.0248 (5)	0.0239 (5)	−0.0019 (4)	0.0021 (4)	−0.0029 (4)
C8	0.0192 (5)	0.0293 (6)	0.0381 (7)	−0.0004 (5)	0.0049 (5)	−0.0012 (5)

### Geometric parameters (Å, º)

**Table:**

O1—C1	1.3157 (13)	C5—H5B	0.9800
O1—C3	1.4629 (13)	C5—H5C	0.9800
N1—C1	1.2981 (14)	C6—H6A	0.9800
N1—C5	1.4658 (14)	C6—H6B	0.9800
N1—C6	1.4707 (15)	C6—H6C	0.9800
C1—C2	1.4848 (15)	S1—O3	1.4390 (9)
C2—H2A	0.9800	S1—O5	1.4433 (9)
C2—H2B	0.9800	S1—O4	1.4440 (9)
C2—H2C	0.9800	S1—O2	1.6175 (9)
C3—C4	1.5001 (18)	O2—C7	1.4356 (14)
C3—H3A	0.9900	C7—C8	1.5079 (17)
C3—H3B	0.9900	C7—H7A	0.9900
C4—H4A	0.9800	C7—H7B	0.9900
C4—H4B	0.9800	C8—H8A	0.9800
C4—H4C	0.9800	C8—H8B	0.9800
C5—H5A	0.9800	C8—H8C	0.9800
			
C1—O1—C3	119.31 (9)	N1—C5—H5C	109.5
C1—N1—C5	120.66 (10)	H5A—C5—H5C	109.5
C1—N1—C6	122.44 (10)	H5B—C5—H5C	109.5
C5—N1—C6	116.88 (9)	N1—C6—H6A	109.5
N1—C1—O1	115.55 (10)	N1—C6—H6B	109.5
N1—C1—C2	123.23 (10)	H6A—C6—H6B	109.5
O1—C1—C2	121.22 (10)	N1—C6—H6C	109.5
C1—C2—H2A	109.5	H6A—C6—H6C	109.5
C1—C2—H2B	109.5	H6B—C6—H6C	109.5
H2A—C2—H2B	109.5	O3—S1—O5	114.46 (6)
C1—C2—H2C	109.5	O3—S1—O4	114.95 (6)
H2A—C2—H2C	109.5	O5—S1—O4	113.02 (6)
H2B—C2—H2C	109.5	O3—S1—O2	101.21 (5)
O1—C3—C4	106.41 (9)	O5—S1—O2	105.75 (6)
O1—C3—H3A	110.4	O4—S1—O2	105.84 (5)
C4—C3—H3A	110.4	C7—O2—S1	116.46 (7)
O1—C3—H3B	110.4	O2—C7—C8	107.41 (10)
C4—C3—H3B	110.4	O2—C7—H7A	110.2
H3A—C3—H3B	108.6	C8—C7—H7A	110.2
C3—C4—H4A	109.5	O2—C7—H7B	110.2
C3—C4—H4B	109.5	C8—C7—H7B	110.2
H4A—C4—H4B	109.5	H7A—C7—H7B	108.5
C3—C4—H4C	109.5	C7—C8—H8A	109.5
H4A—C4—H4C	109.5	C7—C8—H8B	109.5
H4B—C4—H4C	109.5	H8A—C8—H8B	109.5
N1—C5—H5A	109.5	C7—C8—H8C	109.5
N1—C5—H5B	109.5	H8A—C8—H8C	109.5
H5A—C5—H5B	109.5	H8B—C8—H8C	109.5
			
C5—N1—C1—O1	−0.51 (15)	C1—O1—C3—C4	168.71 (10)
C6—N1—C1—O1	177.86 (10)	O3—S1—O2—C7	174.76 (9)
C5—N1—C1—C2	−179.82 (10)	O5—S1—O2—C7	−65.61 (9)
C6—N1—C1—C2	−1.45 (17)	O4—S1—O2—C7	54.55 (9)
C3—O1—C1—N1	178.90 (9)	S1—O2—C7—C8	−178.39 (8)
C3—O1—C1—C2	−1.77 (15)		

### Hydrogen-bond geometry (Å, º)

**Table:**

*D*—H···*A*	*D*—H	H···*A*	*D*···*A*	*D*—H···*A*
C5—H5*B*···O5^i^	0.98	2.32	3.288 (2)	170
C2—H2*A*···O3^ii^	0.98	2.40	3.341 (2)	161
C3—H3*A*···O5	0.99	2.50	3.121 (2)	120
C5—H5*A*···O2^iii^	0.98	2.51	3.372 (2)	147
C6—H6*B*···O5^i^	0.98	2.54	3.453 (2)	154
C4—H4*B*···O2^iv^	0.98	2.55	3.452 (2)	154

Symmetry codes: (i) *x*, *y*+1, *z*; (ii) −*x*, *y*+1/2, −*z*+1/2; (iii) *x*, −*y*+3/2, *z*+1/2; (iv) *x*, −*y*+1/2, *z*+1/2.
