# Supplementary figures and images for: Crystal structure of (eth­oxy­ethyl­idene)di­methyl­aza­nium ethyl sulfate
Source: Acta Crystallogr E Crystallogr Commun. 2015 Nov 7;71(Pt 12):o916. doi: 10.1107/S2056989015020678 (PMC4719878; doi:10.1107/S2056989015020678)

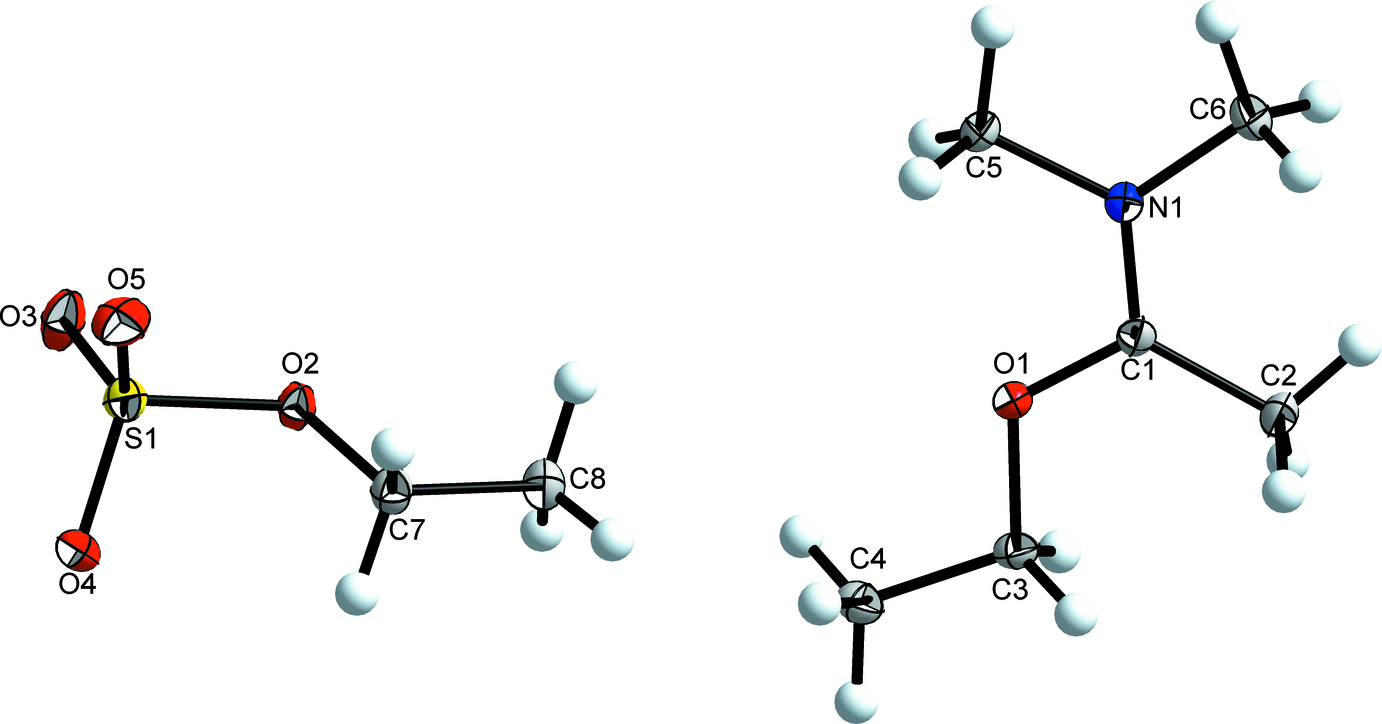

Supplement: Supplementary file 4 [file e-71-0o916-fig1.tif]

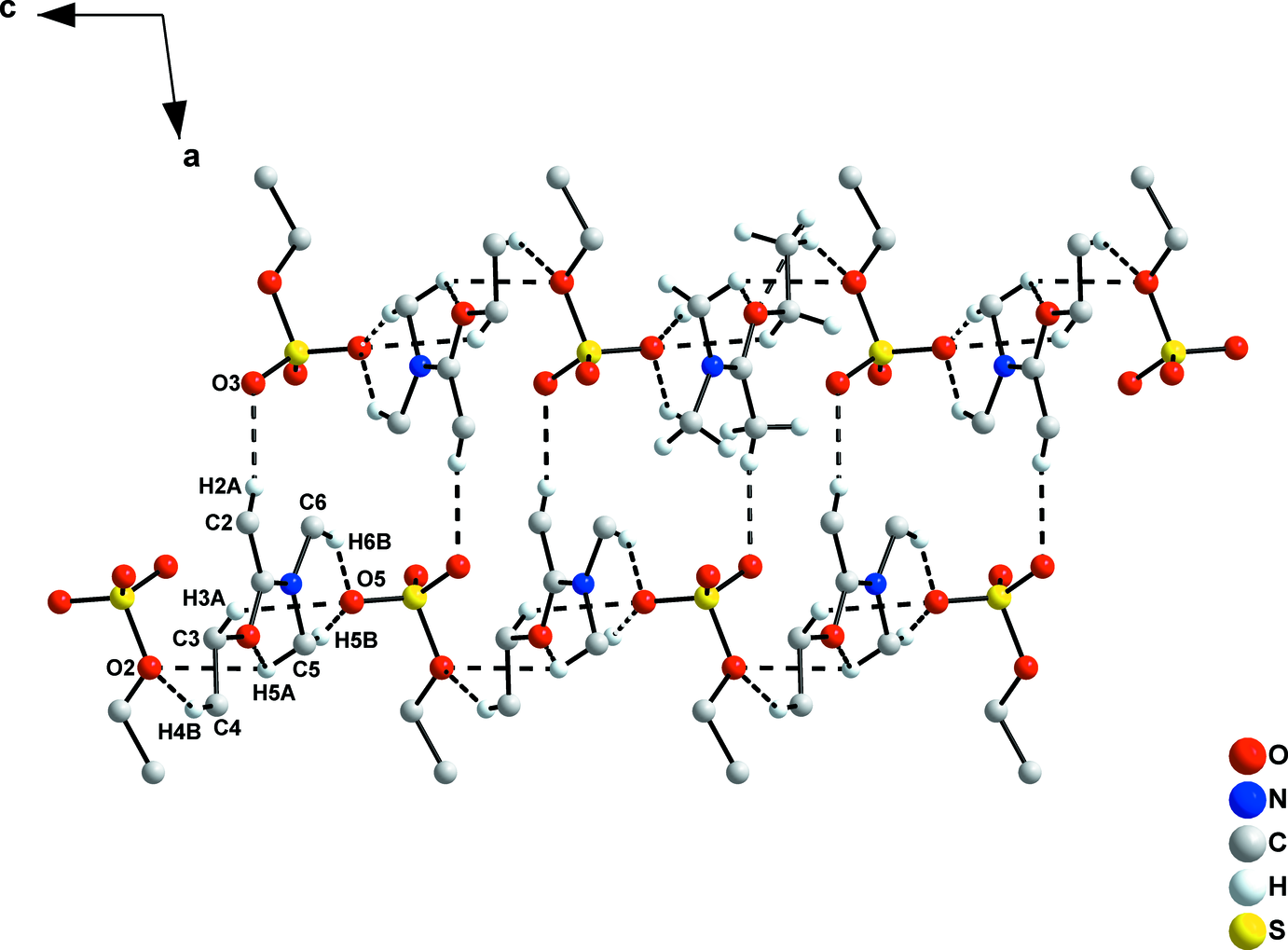

Supplement: Supplementary file 5 [file e-71-0o916-fig2.tif]
